# Nonlinear time series analysis of state-wise COVID-19 in Malaysia using wavelet and persistent homology

**DOI:** 10.1038/s41598-024-79002-0

**Published:** 2024-11-11

**Authors:** Piau Phang, Carey Yu-Fan Ling, Siaw-Hong Liew, Fatimah Abdul Razak, Benchawan Wiwatanapataphee

**Affiliations:** 1 Faculty of Computer Science and Information Technology, Universiti Malaysia Sarawak, 94300 Kota Samarahan, Sarawak Malaysia; 2 Department of Mathematical Sciences, Faculty of Science and Technology, Universiti Kebangsaan Malaysia, 43600 Bangi, Selangor Malaysia; 3 School of Electrical Engineering, Computing and Mathematical Science, Curtin University, Perth, WA 6845 Australia

**Keywords:** Time series analysis, COVID-19, Persistent homology, Topological features, Wavelet, Applied mathematics, Computational science, Infectious diseases

## Abstract

The nonlinear progression of COVID-19 positive cases, their fluctuations, the correlations in amplitudes and phases across different regions, along with seasonality or periodicity, pose challenges to thoroughly examining the data for revealing similarities or detecting anomalous trajectories. To address this, we conducted a nonlinear time series analysis combining wavelet and persistent homology to detect the qualitative properties underlying COVID-19 daily infection numbers at the state level from the pandemic’s onset to June 2024 in Malaysia. The first phase involved investigating the evolution of daily confirmed cases by state in the time-frequency domain using wavelets. Subsequently, a topological feature-based time series clustering is performed by reconstructing a higher-dimensional phase space through a delay embedding method. Our findings reveal a prominent 7-day periodicity in case numbers from mid-2021 to the end of 2022. The state-wise daily cases are moderately correlated in both amplitudes and phases during the Delta and Omicron waves. Biweekly averaged data significantly enhances the detection of topological loops associated with these waves. Selangor demonstrates unique case trajectories, while Pahang shows the highest similarity with other states. This methodological framework provides a more detailed understanding of epidemiological time series data, offering valuable insights for preparing for future public health crises.

**Supplementary Information:**

The online version contains supplementary material available at 10.1038/s41598-024-79002-0.

## Introduction

Since the onset of the COVID-19 pandemic, epidemiological time series data, particularly daily new confirmed case figures, have emerged as crucial yet accessible sources of information for understanding the spatiotemporal dynamics of the outbreak. However, the analysis of these datasets presents various challenges. The nonlinear progression of case numbers^1^, alongside fluctuations and correlations in case amplitudes and phases across different regions^2^, complicates the pictures. Moreover, factors such as seasonality^3^, periodicity^4^ and the occurrence of multiple pandemic waves^5,6^ add layers of complexity to the analysis. These aspects obstruct a comprehensive examination of the pandemic, affecting both the identification of frequent patterns and the recognition of rare or dynamic changes throughout the pandemic.

With COVID-19 likely to remain endemic, it is essential to analyze daily infection number data. This involves generating summaries, identifying similarities and detecting anomalous trajectories through time series analysis. Although various quantitative methods exist, from basic summary statistics and spectral analysis^7^ to complex statistical models fitting such as autoregressive moving average models^8^, there is a notable scarcity of research utilizing topological approaches for discerning qualitative changes in COVID-19 daily case data. Topology, known for its resilience to noise^9^, allows for the exploration of time series dynamics by examining the shape of the data when represented in a higher dimensional data cloud. Topological data analysis (TDA) specially focuses on analyzing the ‘shape’ of high-dimensional datasets without reducing their dimensions^10^. A prime example of this is identifying periodic behavior, which is characterized by data paths that loop back on themselves, thus forming topological circles or loops in the data cloud^11,12^. This approach provides a unique perspective on data analysis, potentially revealing new insights into the behavior of the pandemic.

Within the domain of epidemiological time series, TDA has been effectively used for a qualitative analysis of various infectious diseases. It has provided insights into the peak and dramatic changes in influenza incidence^13^, the spreading dynamics of the Zika virus^14,15^ and also COVID-19^16–18^. Nevertheless, there are certain limitations in the current literature. For instance, the study by Chen and Volić^16^ employs a mapper algorithm, a TDA tool which is not specifically designated for detecting periodicity in time series data. This represents a potential gap in utilizing TDA for comprehensive analysis of COVID-19 data. Additionally, later studies such as those by Ault and Lu^17^ and Hu^18^, did not find significant topological features in their data, which focused on new deaths versus new cases and forecasting, respectively. This highlights the need for further exploration and refinement in applying TDA to harness its potential in understanding the dynamics of COVID-19 and other epidemiological data.

Hence, this study aims to analyze the state-wise COVID-19 daily new case data in Malaysia, spanning from the onset of the pandemic to June 1, 2024, using a combination of wavelet and topological data analysis. Initially, wavelet analysis for examining the potential frequency changes and localized periodic behavior^1^ of time series data is conducted. This method allows for a nuanced examination of the data’s cyclical fluctuations and time-frequency characteristics, as demonstrated in^19^. Subsequently, a topological feature-based approach for time series clustering is performed. This involves reconstructing a higher-dimensional phase space using the delay embedding method. Following this, we extract topological features that encapsulate the shape and inherent patterns within the data.

This two-pronged analytical approach enables a comprehensive understanding of the COVID-19 trends and variances across different Malaysian states. Wavelet analysis is required to examine the strength and time range of synchronization in phase and magnitude of the prominent waves across Malaysian states. Then, we hypothesized that such successive pandemic waves would emerge as periodic orbits in a phase space reconstruction plot and could be captured using persistent homology in TDA.

## Methods

### Wavelet

Wavelet analysis is conducted to examine the localized periodic behavior and probe into the potential frequency changes over time on the spread of state-wise COVID-19 in Malaysia. Specifically, a wavelet phasor mean field magnitude plot is constructed to depict the time (and frequency) dependence of phase synchrony among 13 states and three federal territories COVID-19 daily cases, while the wavelet mean field is computed to study the fluctuations and correlations in both amplitudes and phases.

### Phase space reconstruction

The one-dimensional time series data is reconstructed into higher-dimensional data cloud by applying an embedding technique. Time series embedding refers to a type of transformation ψ: R → R^*m*^ that augments the dimension of the time series using observed values across some time window [*t*_1_, *t*_2_]. Embedding methods are commonly used to analyze nonlinear time series whose entire system state cannot be fully or directly observed^20^. For instance, the dynamics of a one-dimensional time series can be visualized in two- or three-dimensional phase space reconstruction. The set of points in phase space may represent various possible states toward which the system tends to evolve, or even exhibit attractors. The type of dynamics (such as fixed points, or periodic dynamics) can be deduced from the topology of attractors.

One approach to embedding a time series is to consider its data with the time delay version of itself^21^, known as Takens embedding theorem. In this theorem, two parameters, namely the time delay, *τ*, and the embedding dimension, *m*, must be carefully selected. The time delay parameter can be chosen based on the autocorrelation function, average mutual information (AMI) or correlation integral^22^. Instead of employing the autocorrelation function which is a linear statistic, we utilize AMI to take into account nonlinear correlation for estimating an appropriate time delay. Specifically, we select the time lag at which the AMI decays to 1/exponent of its value at zero as *τ*. The delayed choice could be linked to the memory and characteristics of the time series dynamics, such as the incubation period and quarantine window in the contexts of epidemiology.

**Fig. 1:**
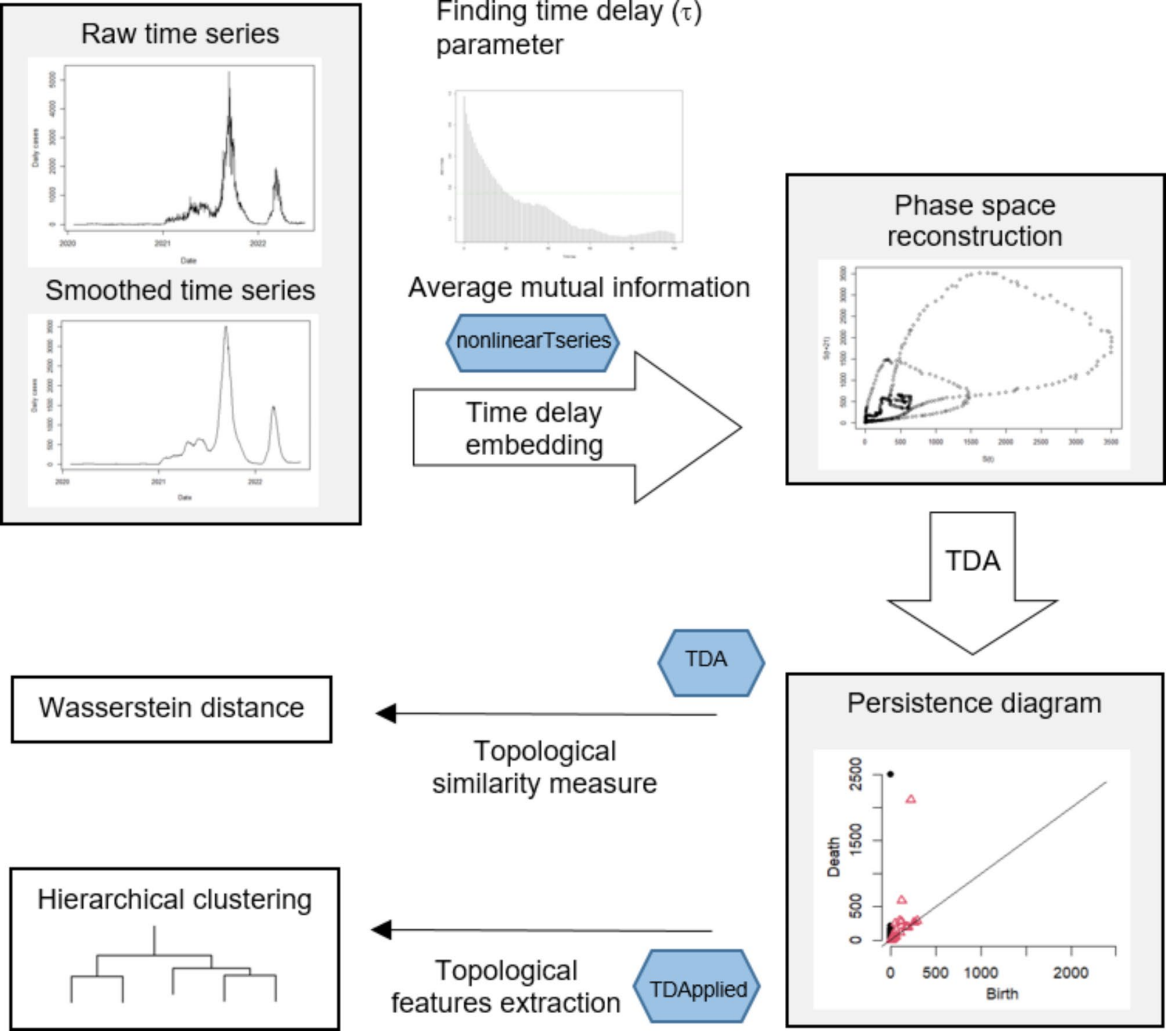
A summary of the methodology pipeline for comparing the state-wise time series data using topological data analysis. Blue hexagon symbols highlight the R package used in this study.

On the other hand, the embedding dimension, *m*, implies that the time series has to be plotted against itself using the delay, *τ* for *m* − 1 number of times^23^. For instance, 3-dimensional embedding implies that the delay vector $\:\overrightarrow{x}\left(t\right)=(x\left(t\right),x\left(t+\tau\right),\:x\left(t+2\tau\right))$ is constructed. Since it is not feasible to visualize multi-dimensional data clouds that are higher than three dimensions, we select *m* = 2 to simplify our analysis. Also, as optimal embedding often does not exist in stochastic systems^24^, we apply a rolling average to reduce noise and smooth the COVID-19 time series data. A summary of the methodology pipeline used in this study is given in Fig. 1.

### Topological data analysis

The number, characteristics and persistence of structural features in the 2-dimensional data point cloud created in the phase space reconstruction are investigated through persistent homology. Persistent homology is a method in TDA that captures the changing topological features or shape structure of a space (such as point cloud, image, etc.) over different spatial resolutions. Many infectious disease time series have a cyclical element. Hence, another tool in TDA, namely the Mapper algorithm, is not used in this study since it is not designed to detect periodic structures in the data cloud.

**Fig. 2:**
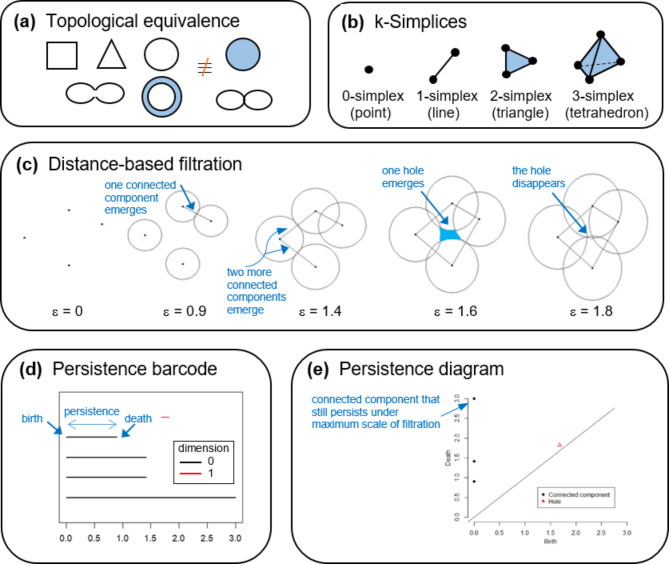
A schematic overview of TDA and persistent homology.

A schematic overview of TDA and persistent homology is given in Fig. 2. Two geometries are topologically equivalent if one object can be reshaped into another without cutting or gluing. Topological features are represented as a simplicial complex, which is a set composed of points, lines, triangles, tetrahedrons and other higher-dimensional counterparts. A filter metric, for instance, distance values, is used to partition the data cloud into overlapping bins for generating a series of simplicial complexes through a filtration process. Starting from 0-simplex (vertex, point) and for an increasing sequence of filter metric values, 1-simplex (edge, line segment), 2-simplex (triangle), and 3-simplex (tetrahedron) etc. enter the sequence. To illustrate the persistence (i.e. the difference between birth time and death time) of multi-scale topologies features, we can plot the persistence barcode and persistence diagram. In this study, the topological features of interests include connected component (0-dimensional feature) and loop, hole or ring structure (1-dimensional feature). The emergence of periodic or recurrent behavior in time series data is characterized by the path returning to itself, producing a 1-dimensional loop in the embedding space^11^.

To compare the topological features of state-wise time series of COVID-19 cases throughout the study period, we measure the similarity between persistence diagrams for any two states using Wasserstein distance. The Wasserstein distance is computed by matching all points (either 0-dimensional or 1-dimensional features) in two persistence diagrams. A relatively lower Wasserstein distance means that the two data clouds are topologically similar or share enough topological features of similar size and persistence.

Apart from that, the topological features of each state’s persistence diagram are used to perform hierarchical clustering to further categorize the progression of state-wise COVID-19 in Malaysia into several clusters. Four features are selected for each state. These topological features include the appearance of one remaining connected component for all data points in phase space reconstruction, the maximum (i.e. longest) lifetime of a one-dimensional loop, the second longest lifetime of a one-dimensional loop and the total persistence (i.e. the sum of all lifetimes) of one-dimensional loops. In 0-dimension, there is always a connected component that never dies. The birth of such a connected component is marked by the second-highest black dot on the death axis (y-axis), whereas the black dot at the top-left corner of the persistence diagram signifies that the connected component still persists under the maximum scale of the filtration.

The topological-based clustering is validated by comparing their results with those obtained from hierarchical clustering on principal components as there is no ground truth cluster labelling for state-wise COVID-19 data. Following^25^, three performance metrics are used, namely the rand index (RI), normalized mutual information (NMI), and normalized variation of information (NVI). All these three metrics range from 0 to 1. For RI and NMI, 1 indicates identical or good clustering while 0 implies the opposite. They respectively measure the similarity and amount of information shared between two clustering results. Meanwhile, NVI measures the dissimilarity between two clustering results based on information theory. Lower NVI values suggest a smaller difference (i.e. better agreement) between two clustering results.

## Results

### Wavelet analysis

**Fig. 3:**
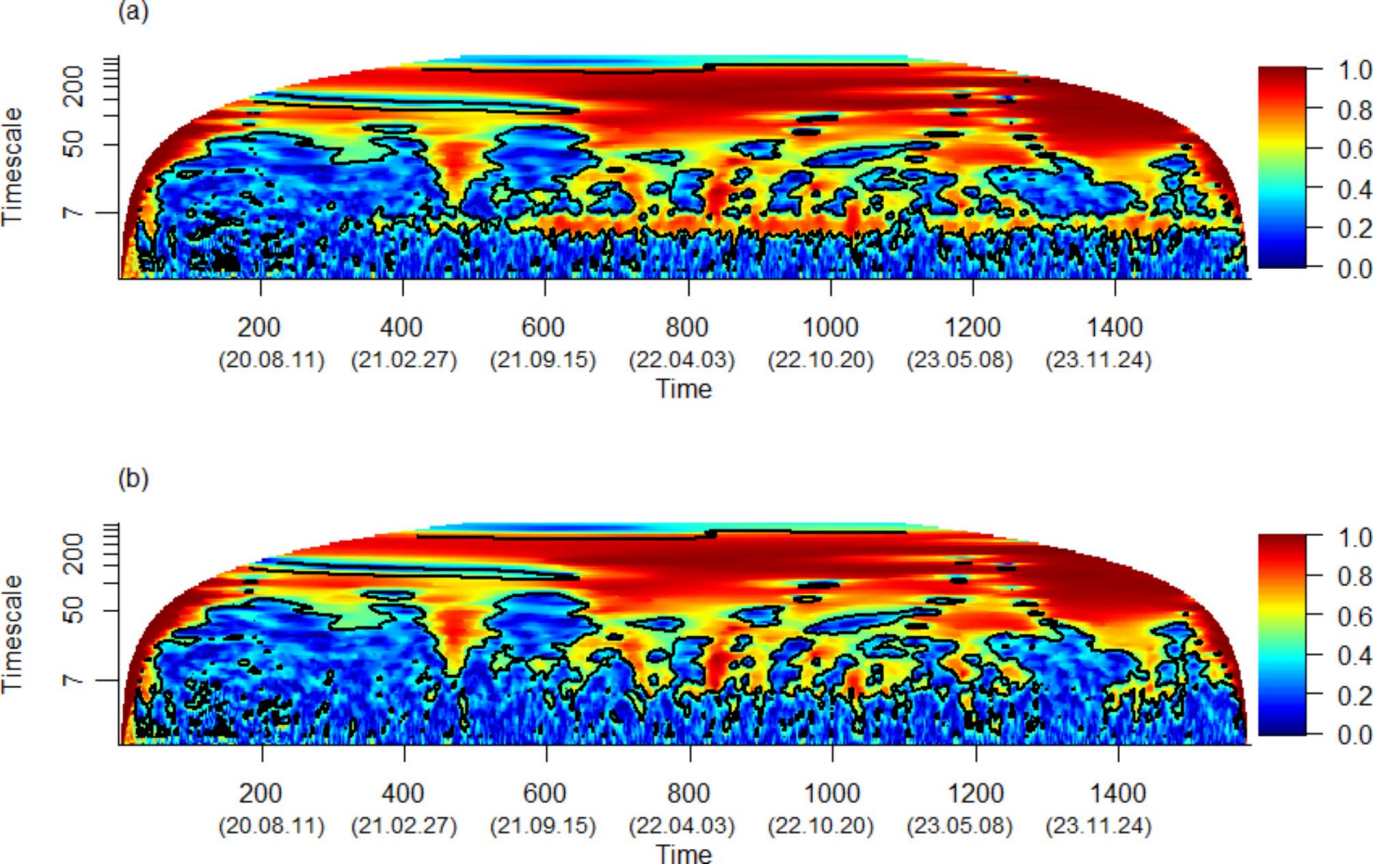
The wavelet phasor mean field magnitude plot for state-wise COVID-19 daily cases (**a**) raw time series, and (**b**) 7-day rolling average, from the onset of the pandemic up to June 1, 2024.

Our analysis first explores the phase synchronization strength across all Malaysian states and federal territories concerning daily COVID-19 cases during the study period. This is achieved by constructing a wavelet phasor mean field magnitude plot, as given in Fig. 3(a). In this plot, when a group of time series exhibits the same phase, their corresponding phasors align in similar directions on the complex plane, resulting in the summation of a large magnitude complex number. This visualization technique is particularly effective in identifying synchronized cycles. Notably, in Fig. 3(a), synchronous cycles with a period of 7 days are evident, especially from mid-2021 to end of 2022. This observation suggests a rapid periodicity or could indicate weekly reporting patterns in COVID-19 case data, a phenomenon also reported in global studies^1,26,27^. These findings provide valuable insights into the temporal dynamics of COVID-19 case reporting across Malaysia.

Applying a 7-day rolling average to smooth the daily COVID-19 case data results in a notable change in the observed patterns, as depicted in Fig. 3(b). Specifically, this smoothing technique reduces the prominence of the 7-day periodicity, while the phase synchronization at higher frequency bands (i.e. timescale greater than seven days) remains unchanged. This indicates that, when examined over a longer duration, such as spans of 50 days or more, the 7-day rolling average of COVID-19 confirmed cases across all states and federal territories in Malaysia demonstrates a high degree of synchronization throughout the study period. In the medium timescale range between 10 and 49.9 days, we observe that the state-wise COVID-19 cases tend to align in the same phase during several key periods: early 2020, April to mid-July 2021, October to November 2021, February to April 2022, and last quarter of both 2022 and 2023. These findings largely agree with existing evidence on seasonal spikes in COVID-19 from November through April^28^ and recurrence waves over a period of three to nine months^3,27^. This analysis suggests varying degrees of synchronicity in the spread of COVID-19 across different timescales and highlights the critical role of timely provision of epidemiological data, offering insights into the temporal dynamics of the pandemic across Malaysia.

**Fig. 4:**
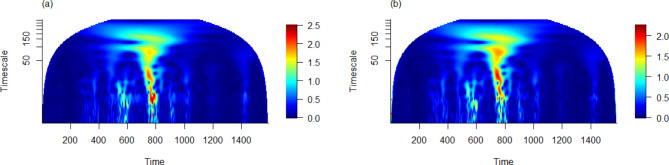
The wavelet mean field magnitude plot for state-wise COVID-19 daily cases (a) raw time series, and (b) 7-day rolling average, from the onset of the pandemic up to June 1, 2024.

While the wavelet phasor mean field in Fig. 3 effectively illustrates phase synchronization in the time series data, it does not account for the magnitude of these series. To address this issue, we constructed wavelet mean field plots in Fig. 4, enabling the identification of scales and time points where both phases and magnitude of state-wise COVID-19 daily cases in Malaysia align consistently. These plots reveal that the daily cases across states are not only moderately correlated in terms of amplitudes and phases, but also exhibit consistent patterns over a relatively shorter duration of time (less than 50 days). Notably, such synchronization is observed from June to mid-September 2021, coinciding with the Delta variant wave, and from February to April 2022, aligning with the Omicron variant waves^29^. Additionally, for longer periods of components ranging from 50 to 250 days, a consistent pattern is evident throughout 2021 and 2022. This comprehensive analysis incorporating both phase and magnitude provides a more holistic view of the temporal dynamics and synchronization patterns of COVID-19 cases across Malaysia.

### Phase space reconstruction

**Fig. 5:**
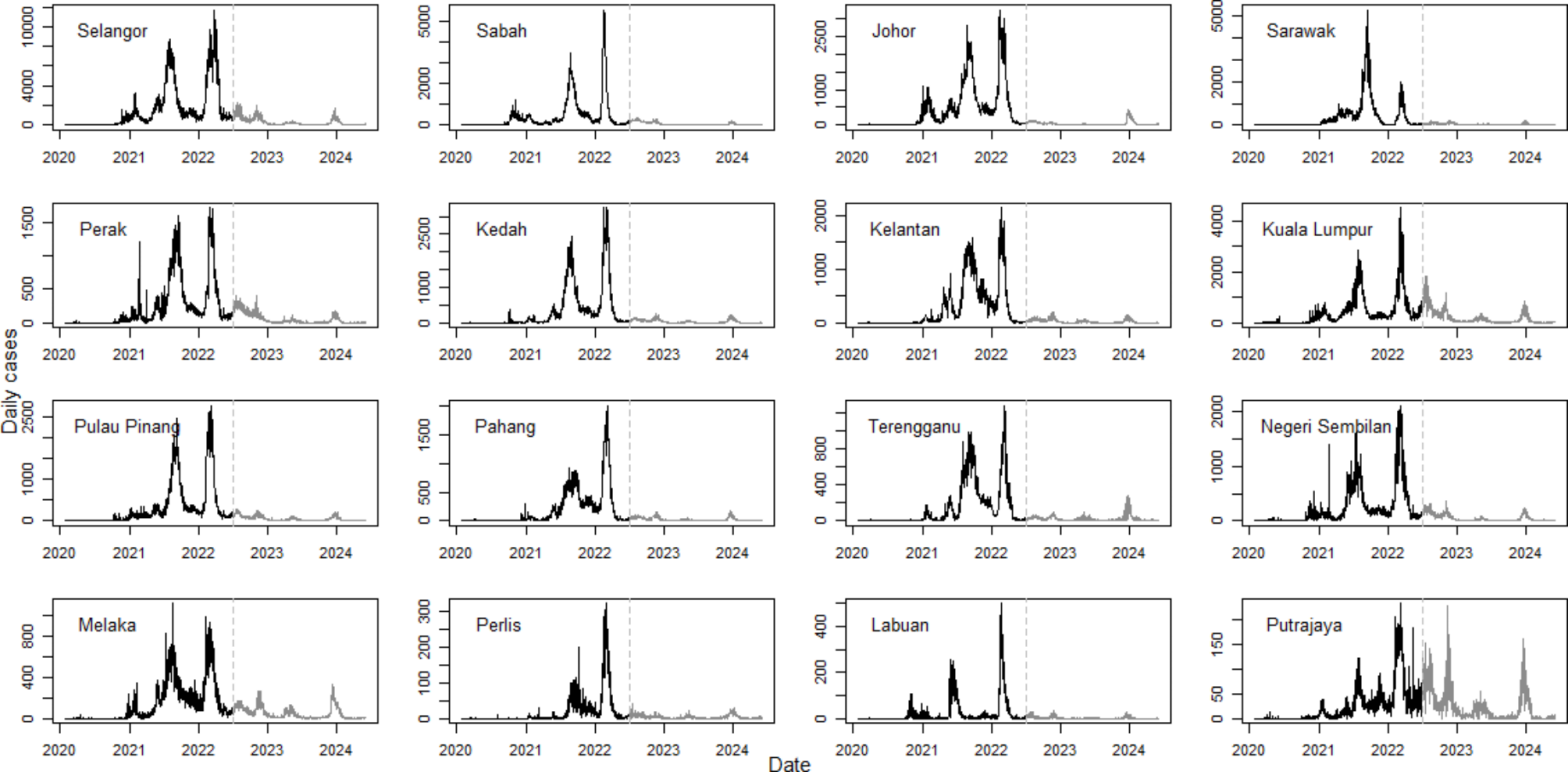
The raw daily confirmed cases of COVID-19 in 13 states and 3 federal territories of Malaysia. The vertical dotted line marks the date of June 30, 2022. See Fig. S1 in Supplementary Information for the daily or cumulative newly confirmed cases for the whole of Malaysia throughout the study period, as well as the geographical map of Malaysia.

Figure 5 depicts a comprehensive visualization of the raw daily confirmed COVID-19 cases across 13 states and three federal territories in Malaysia, spanning from January 25, 2020 to June 1, 2024, providing a clear and up-to-date overview of the pandemic progression across Malaysia. Accompanying this visual representation, Table 1 provides detailed information on each region’s peak daily case count and their respective dates. The trajectory of COVID-19 in all states appears to exhibit at least two significant waves (see the curves to the left of the vertical dotted lines in Fig. 5). The first of these major waves occurred in the latter half of 2021, corresponding with the Delta variant’s prevalence, and the second wave emerged in the first quarter of 2022, during the spread of the Omicron variant. Interestingly, except Sarawak and Melaka, all other regions experienced the highest daily case numbers during the period dominated by the Omicron variant. This trend highlights the distinct impact and progression of different COVID-19 variants across Malaysia’s diverse regions.

Figure 5 also reveals that the spread of COVID-19 has stabilized in most states since the second half of 2022. Notably, only Putrajaya’s line chart shows significant fluctuations for the period after June 2022. However, this unstable pattern does not indicate a new wave or resurgence of the pandemic. Instead, the noticeable spikes are due to the scale of the data. Being a spatial unit with the lowest population size in this study (see Table 1), Putrajaya recorded a relatively lower order of magnitude for overall case count than other states, making its line chart more sensitive to changes.

The transition of COVID-19 from the pandemic to the endemic phase for the whole of Malaysia started on April 1, 2022^30^ as announced by the Ministry of Health Malaysia. The COVID-19 data updates may be limited to a weekly basis in the endemic stage instead of a daily basis in the pandemic stages. Also, similar to the situation in other countries such as the United States^31^, the testing and case reliability in the endemic phase significantly decreased compared to the large-scale case tracing in the first two years of pandemic. Hence, we showcase the phase space reconstruction and subsequent topological features extraction on the time series data from the onset of the pandemic to June 30, 2022, in this section, while the full analysis over the extended time period up to June 1, 2024, is given in the Supplementary Information. Apart from reducing the computational costs, this pandemic phase corresponds to the implementation of most of the restrictive measures, especially the inter-state travel bans throughout Malaysia. Focusing on this pandemic phase data may lead to a more realistic topological-based clustering of the state-wise COVID-19 prevalence in Malaysia.

While the time series data on COVID-19 cases are regularly collected, they are inherently subject to noise and irregular fluctuations. These elements can adversely impact the quality of phase space reconstruction and, consequently, the extraction of subsequent topological features. To mitigate this issue, generating a smoother time series is crucial. This is achieved by applying a rolling average to the data prior to selecting the time delay parameter based on AMI.

Table 1 gives the time delay parameter selected. Without smoothing, the range of time delay selected falls within $\:\:[9,\:19]$, with an exception for Putrajaya. Conversely, when the data is smoothed, the time delay ranges for 7-day and 15-day rolling averages are relatively similar, predominantly within the 16 to 30 range, except for Pahang. This indicates that unsmoothed time series generally require a shorter time delay compared to their smoothed counterpart. A shorter time delay suggests that brief temporal correlations are present in the data, implying that the successive values of the time series amplitude are influenced predominantly by a few preceding values^32^. This distinction between smooth and unsmoothed data is crucial for accurately capturing and interpreting the dynamic patterns within the time series.

**Table 1:** The state population, population density, highest daily cases, and time delay parameter were selected for respective raw daily cases and rolling average cases from the onset of the pandemic up to June 30, 2022.

State or federal territory	Population (2021)	Population density (persons / km^2^)		Time delay (τ)		
			Highest daily cases (Date)	Raw daily cases	7-day rolling average cases	15-day rolling average cases
Selangor	6,815,200	880	11,692 (24 Mac 2022)	11	21	22
Sabah	4,111,700	46	5,565 (18 Feb 2022)	15	17	17
Johor	3,978,700	209	3,238 (12 Feb 2022)	16	29	29
Sarawak	2,946,400	20	5,291 (12 Sept 2021)	12	20	21
Perak	2,632,500	118	1,713 ( 3 Mac 2022)	12	24	25
Kedah	2,298,500	225	3,243 (17 Feb 2022)	16	20	21
Kelantan	1,998,200	119	2,135 (23 Feb 2022)	16	25	23
Kuala Lumpur	1,925,200	8,157	4,527 ( 8 Mac 2022)	9	20	20
Pulau Pinang	1,825,800	1,659	2,773 (10 Mac 2022)	16	18	19
Pahang	1,773,600	44	2,006 (11 Mac 2022)	19	47	50
Terengganu	1,318,800	89	1,283 ( 9 Mac 2022)	17	21	24
Negeri Sembilan	1,172,700	180	2,115 (10 Mac 2022)	14	27	29
Melaka	973,200	583	1,120 (16 Aug 2021)	16	25	30
Perlis	267,400	348	321 (27 Feb 2022)	9	20	22
Labuan	104,400	1,034	499 (23 Feb 2022)	12	16	17
Putrajaya	95,900	2,215	231 (11 Mac 2022)	2	19	20

**Fig. 6:**
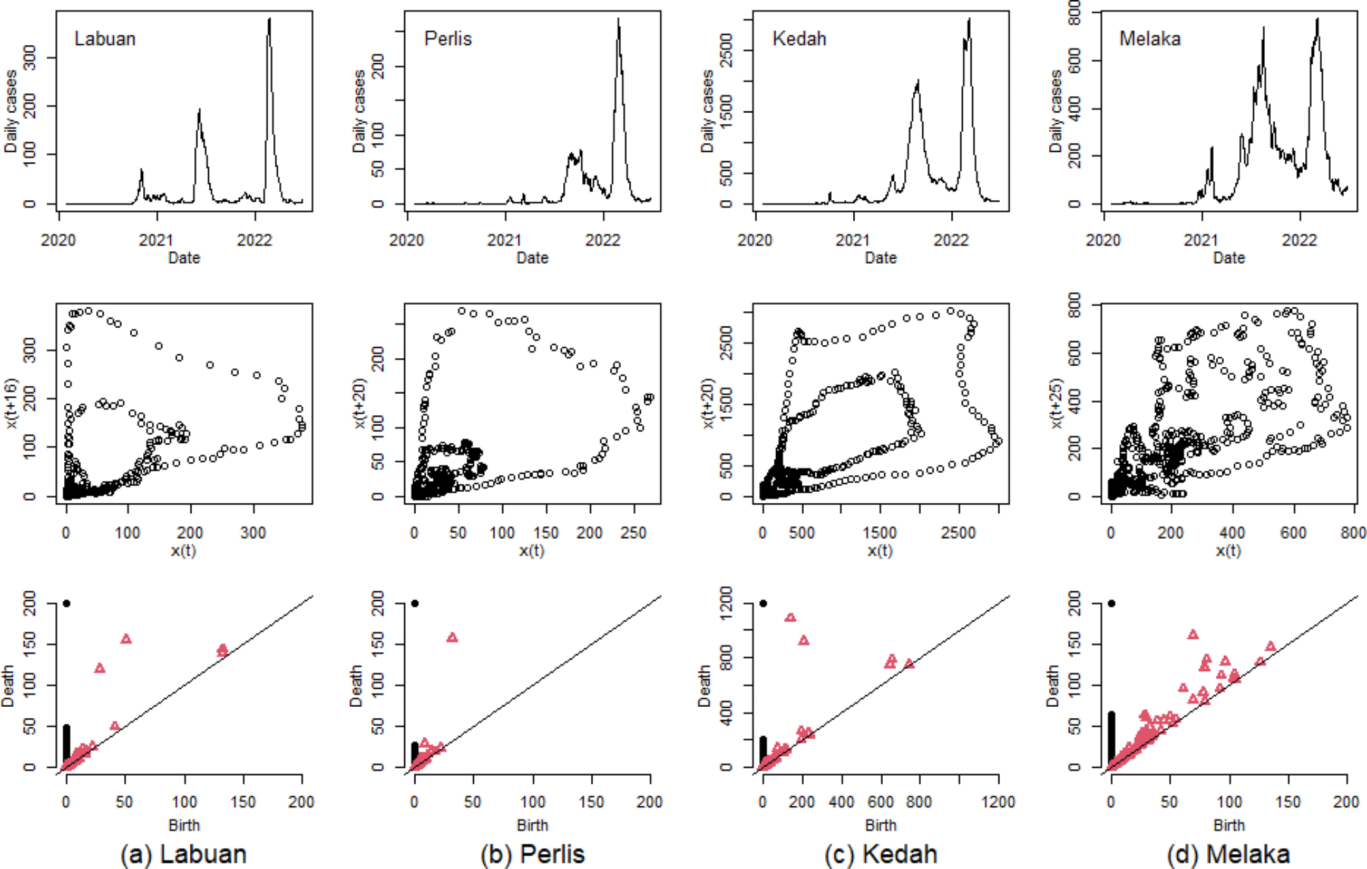
Smoothed time series of COVID-19 daily confirmed cases with 7-day rolling average (first-row panels), phase space reconstruction plot (second-row panels) and persistence diagram (third-row panels) for (**a**) Labuan, (**b**) Perlis, (**c**) Kedah, and (**d**) Melaka. The black dots in the persistence diagram represent 0-dimensional connected components and the red triangles represent 1-dimensional loops.

Figure 6 gives the smoothed time series of COVID-19 daily confirmed cases using a 7-day rolling average for four selected states: Labuan, Perlis, Kedah, and Melaka. Accompanying these time series are their respective phase space reconstruction plots and persistence diagrams. In the cases of Labuan, Perlis, and Kedah, the cyclical behavior evident in the 7-day rolling average time series is effectively captured in their phase space reconstruction plots. For instance, the reconstructed attractor for Labuan clearly shows three circular paths of varying sizes, corresponding to the slow, intermediate and fast dynamics of the three dominant waves in its smoothed time series. The structure is well preserved in the phase space reconstruction, largely due to the ample time intervals between the peaks of consecutive waves in the Labuan time series.

Similarly, the dynamics of the two most dominant waves in the Perlis and Kedah time series are fully captured as outer and inner cycles in their reconstructed phase space. However, the phase space reconstruction for smoothed Melaka time series, using a 7-day rolling average, is notably less effective. This suggests that not all states in Malaysia have COVID-19 daily cases time series that are conducive to high-quality phase space reconstruction using a 7-day rolling average. This variability highlights the importance of tailoring analytical approaches to the specific characteristics of each state’s data.

### Topological data analysis

The emergence of a dominant wave in the COVID-19 time series is characterized by the formation of a 1-dimensional loop within the embedded space, a feature that can be effectively detected using topological approaches, particularly through the construction of a persistence diagram. In the last row of Fig. 6, the persistence diagrams provide a concise summary of the birth and death of both 0-dimensional (connected components, represented by black dots) and 1-dimensional (loops, represented by red triangles) topological features of data clouds. For instance, in the persistence diagrams of Labuan and Kedah, two significant 1-dimensional topological circles or loops are observed (indicated by red triangles positioned further from the diagonal line). These loops correspond to the two most dominant waves in the COVID-19 trajectories of these states. Similarly, the persistence diagram for Perlis reveals a notable red triangle that persists until around 150, indicating the presence of a dominant wave. Additionally, the second most dominant wave is identified by another red triangle, slightly elevated above the cluster of triangles near the diagonal line. These findings demonstrate that with accurate phase-space reconstruction, the oscillatory dynamics of the COVID-19 time series are effectively illustrated through persistent homology, providing a clear and insightful representation of the pandemic’s progression.

However, due to the volatility nature of COVID-19 case time series data, among persistence diagrams constructed for all 13 states and 3 federal territories (refer to Figs. S2-S4 in Supplementary Information), only half of them have the ability to capture the two most dominant waves as significant 1-dimensional topological features. For instance, only one significant loop is visible at (birth, death) coordinates of (70, 160) in the persistence diagram for Melaka in Fig. 6(d). In contrast, other detected 1-dimensional loops may be considered as noise or less significant. This observation suggests that employing a 7-day rolling average, while helpful, may not always be adequate for creating a sufficiently smoothed time series for optimal phase-space reconstruction and accurate extraction of topological features.

**Fig. 7:**
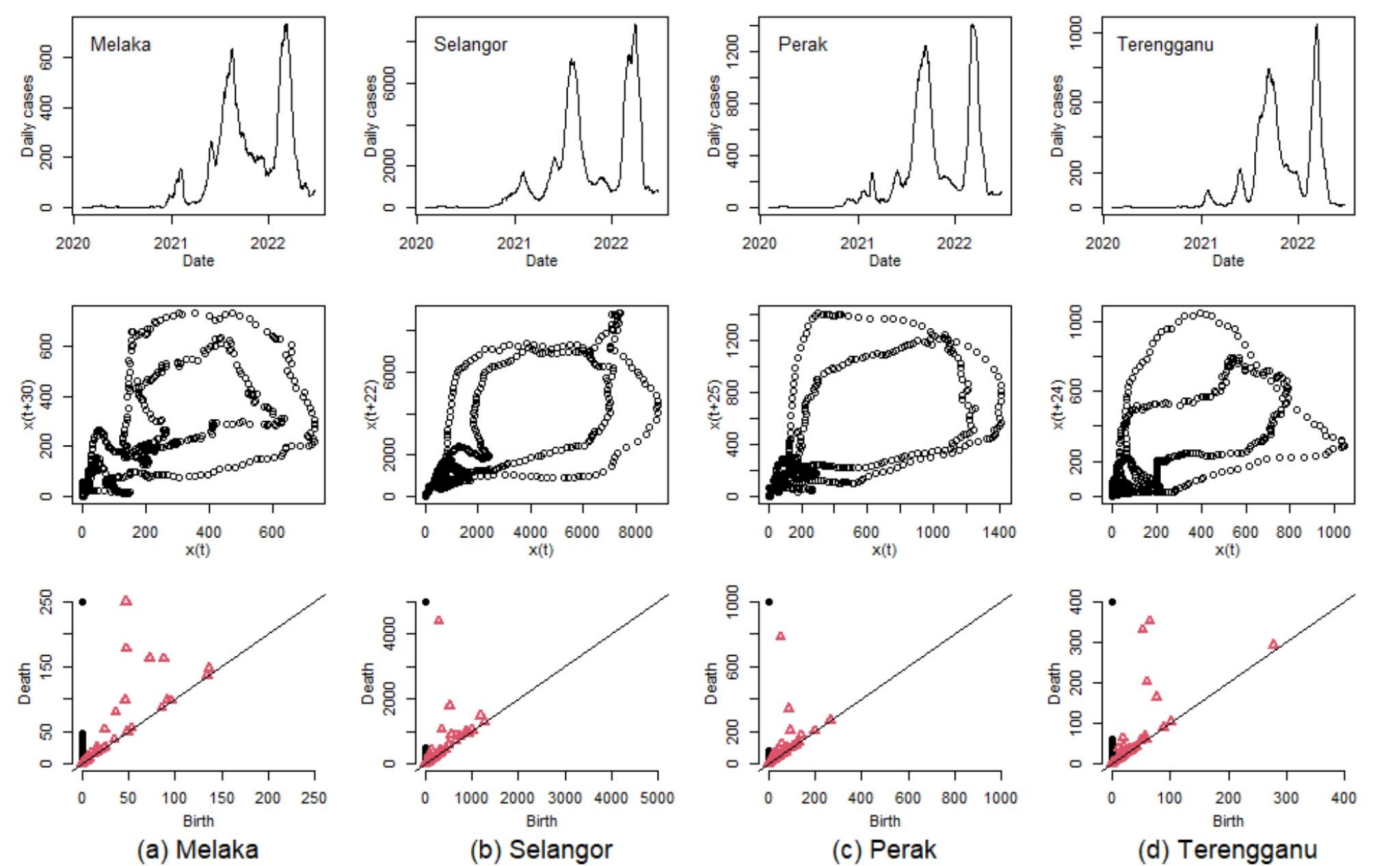
Smoothed time series of COVID-19 daily cases with a 15-day rolling average, phase space reconstruction and persistence diagram for (**a**) Melaka, (**b**) Selangor, (**c**) Perak, and (**d**) Terengganu. The complete set of phase space reconstructions and corresponding persistence diagrams for all 13 states and 3 federal territories from the onset of the pandemic up to June 30, 2022 are available in Figs. S5-S7 of the Supplementary Information.

To further explore the effectiveness of smoothing techniques in time series analysis, Fig. 7 presents examples of phase space reconstruction and persistence diagrams for COVID-19 case time series smoothed with a 15-day rolling average. The phase space reconstruction plots for the four selected states all demonstrate clearly distinguishable inner and outer loops. This is a noteworthy observation, considering the vast differences in the magnitudes of COVID-19 cases across these states. The two most dominant waves in the data are represented as two red triangles positioned far from diagonal lines in the persistence diagrams, as shown in the last row of Fig. 7. These representations highlight the improved clarity and distinction of the pandemic’s major waves in the data, facilitated by the use of a 15-day rolling average. This comprehensive visualization aids a deeper and more nuanced understanding of the temporal dynamics of COVID-19 cases across different regions, showcasing the utility of extended smoothing periods in topological data analysis.

**Fig. 8:**
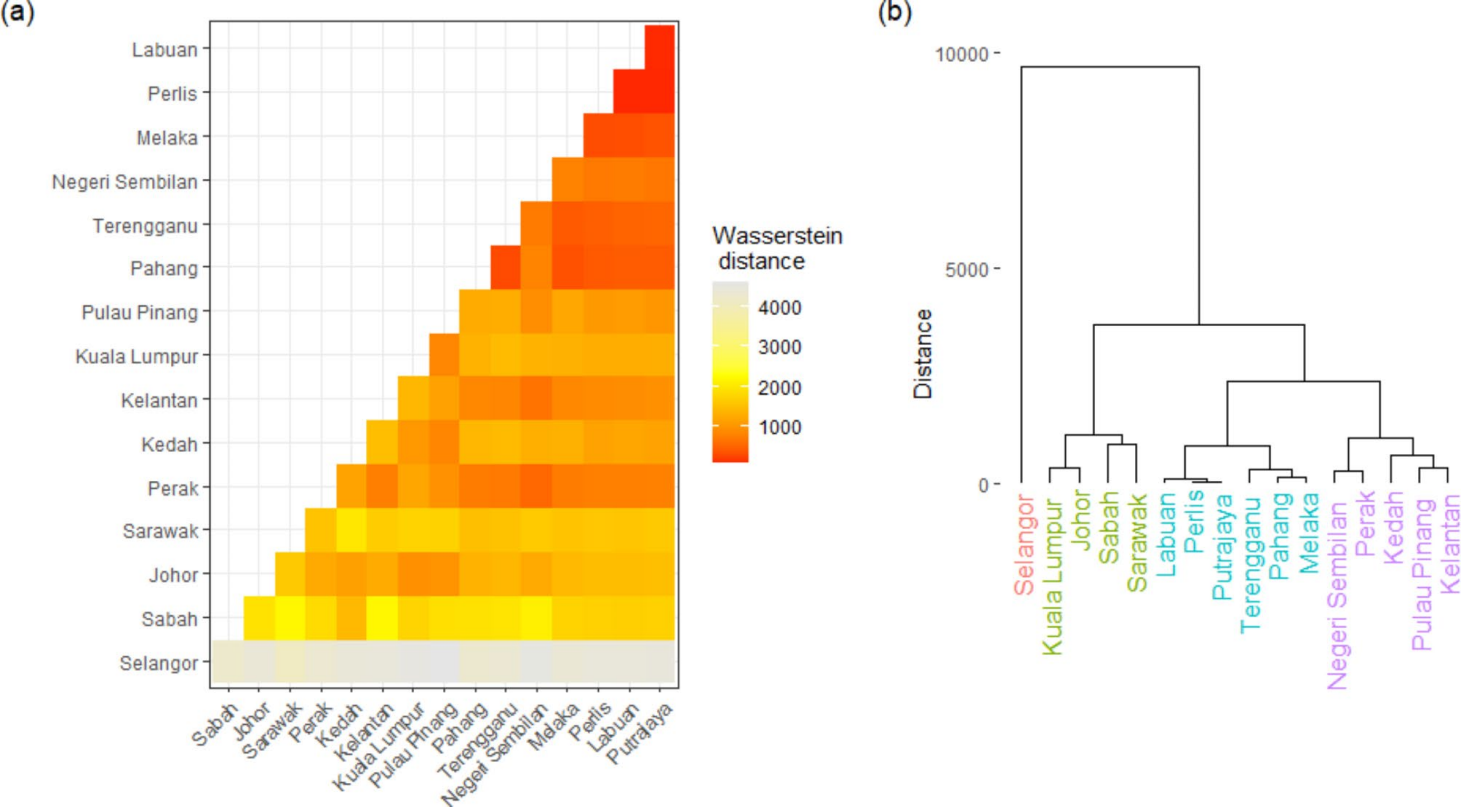
(**a**) Wasserstein distance between persistence diagrams for all 13 states and 3 federal territories. (**b**) The dendrogram of 13 states and 3 federal territories resulting from hierarchical clustering of topological features extracted from their respective persistence diagram.

The pairwise comparison for persistence diagrams of all 13 states and three federal territories in Malaysia, measured using the Wasserstein distance is shown in Fig. 8(a). In this context, a lower Wasserstein distance indicates a higher topological similarity between two persistence diagrams. Selangor, which has the highest population and daily cases recorded (see detailed in Table 1), exhibits a unique trajectory. Consequently, when compared to other states, Selangor’s persistence diagram yields larger Wasserstein distances, highlighting its anomalous nature in the dataset. Conversely, the lowest Wasserstein distances are found at the top-right corner of Fig. 8(a), namely Perlis, Labuan, Melaka and Putrajaya, all of which have relatively small population sizes (see Table 1) despite geographically not adjacent. This observation might suggest a strong positive correlation between the total population and COVID-19 case numbers, a relationship also identified in the study by Wong et al.^33^. Additionally, the persistence diagram of Pahang shows high topological similarity with several other states, possibly due to its geographical position, sharing borders with six states in Peninsular Malaysia. This geographic proximity might also explain why the optimal time delays for Pahang’s 7- and 15-day rolling averages are exceptionally high (47 and 50, respectively, as mentioned in Table 1), reflecting the interconnected dynamics of COVID-19 spread in these adjacent regions.

Figure 8(b) presents the hierarchical clustering of topological features derived from the persistence diagrams of state-wise COVID-19 daily confirmed cases in Malaysia, covering the period from January 25, 2020 to June 30, 2022 in Malaysia. This analysis reveals that the COVID-19 cases across the Malaysian states and federal territories can be broadly grouped into four distinct clusters based on their topological similarities. Selangor stands out as being topologically dissimilar from the other states, likely due to its unique characteristics such as having the highest population and COVID-19 case counts, as detailed in Table 1. Another cluster includes two states from East Malaysia (Sabah and Sarawak) along with Johor and Kuala Lumpur. These regions are characterized by high population, suggesting a possible link between population size and COVID-19 spread patterns.

Additionally, the dendrogram in Fig. 8(b) shows clusters on the right side and center, composed of states with intermediate and low population sizes, respectively (as indicated in Table 1). This clustering pattern further supports the notion that population size significantly influence the spread of COVID-19. The hierarchical clustering thus provides a visual and analytical framework to understand how demographic factors, like population size, have impacted the pandemic’s trajectory across different regions in Malaysia.

**Table 2:** The RI, NMI, and NVI for clustering validation between the hierarchical clustering of topological features and principal components.

Rolling average	Principal component (PC)	Cumulative variance (%)	Clustering comparison measures		
			RI	NMI	NVI
15-day	PC1	65.392	0.800	0.757	0.391
	PC2	75.946	0.792	0.668	0.501
	PC3	85.090	0.550	0.400	0.757

Even though the hierarchical clustering in Fig. 8(b) is obtained by measuring the dissimilarities between features in state’s persistence diagrams and based only on four selected values of topological features, the performance of this topological-based clustering is comparable with the hierarchical clustering on the principal components of the whole data for the respective study period (see Fig. S8 in Supplementary Information). From Table 2, the RI and NMI for PC1 are both higher than 0.75, and its NVI is less than 0.4, indicating the topological-based clustering is able to give result that is almost identical to the clustering based on first principal component, which explains 65% of the data variance. However, the clustering comparison results for PC2 and PC3 are not as promising as those associated with PC1. This may be partially due to the fact that the topological-based clustering performed using features largely relies on the periodicity of COVID-19 recurrent outbreaks. The complexity of such recurrent waves is linked with the concurrent role of containment measures, viral gene mutations, and vaccination programs^34^, which are other factors not being considered in this study for selecting clusters to ensure its validity and rationality^35^.

## Discussion

In this study, we have applied wavelet transform plots and persistence diagrams to conduct an in-depth analysis of daily new confirmed COVID-19 cases. Whilst these methods are well established in other fields, their innovative application to public health data represents a significant novelty in our approach. Our approach offers a detailed exploration of the evolution of COVID-19 pandemic at the state level in Malaysia from January 25, 2020 to June 1, 2024. It enables us to identify states with similar or anomalous pandemic trajectories by describing the topological features in the time series data, following a persistent-homology-based machine learning pipeline as suggested by Pun et al.^36^.

To enhance the clarity of our analysis, we mitigated data noise by applying rolling averages. Specifically, the use of 7- and 15-day rolling averages - effectively weekly and biweekly averages - allowed for a clearer visualization of COVID-19 trends by smoothing out the irregularities and granularity inherent in daily reporting, such as the typical weekend reporting dips^37^. This smoothing process is very pertinent as not only the quality of phase space reconstructed but also the performance of persistent homology degrades rapidly under increasing noise^38^ or intricate shape patterns (e.g. doubled peaked) in time series^39^. As all the state-wise COVID-19 data that we analyzed exhibit a minimum of two peaks (for Delta and Omicron waves respectively), the emergence of these oscillations can be well-captured by the birth of 1-dimensional loops (or cycles) in the embedded space. By finding their persistence, such fluctuations can be further split into inner and outer cycles, which allows us to capture the slow and fast dynamics^40^ of the disease’s prevalence.

As presented in Table 1, Pahang exhibits an exceptionally large optimal time delay across the raw, 7-day, and 15-day rolling average schemes. A large time delay often indicates a more unstable and oscillatory behavior in a time series^41,42^. Figure S7 in Supplementary Information further supports this finding, showing that the persistence diagrams for Pahang and Melaka (i.e. the states with the largest and second-largest time delays in the 15-day rolling average scheme, see Table 1) exhibit more 1-dimensional loops with intermediate lifetimes compared to other states, and these loops occur on similar scales. Mansor^43^ reported that Pahang and Melaka had an identical number of COVID-19 infection clusters in 2021, while Chin^44^ observed that the patterns of shorter waves, aside from the Delta and Omicron waves, were similar in these two states. This suggests that the magnitude of the time delay offers an additional perspective for clustering time series with similar oscillatory behavior and stability.

Furthermore, the analysis of phase synchronization and topological similarity among the states and federal territories, as depicted in the Wasserstein distance comparisons and hierarchical clustering, unveils critical insights into the inter-regional spread and control of COVID-19 in Malaysia. Despite having the largest optimal time delay, Pahang was found to show greater topological similarity with several other states (see Fig. 8(a)). Even though Pahang is the largest state by land area in Peninsular Malaysia and shares borders with six other states, Pahang was the most effective state in managing the COVID-19 outbreak in Malaysia^45^. In the early stage of the pandemic, Pahang took longer days to experience an exponential rise in the number of COVID-19 cases^46^. The geographical centrality of Pahang within Peninsular Malaysia (see Fig. S1(c)), on the one hand, may have a high impact on the inter-state disease transmission and subsequently lead to greater topological similarity in Pahang’s persistence diagram compared with other states’. On the other hand, such geographical proximity and greater inter-state spread may also cause the time series of COVID-19 cases in Pahang to become more oscillatory and unstable, eventually giving rise to a higher optimal time delay.

The distinctive trajectory of Selangor, due to its high population size and case numbers, compared to other states, underlines the unique challenges faced by densely populated areas. This observation is corroborated by the clustering patterns that align closely with population size^47,48^ and geographic proximity^49^. Such findings highlight the significant influence of demographic and geographic factors^50^ on the pandemic’s spread, underscoring the need for tailored public health strategies that consider these regional characteristics. For instance, densely populated regions may require more stringent containment measures and robust healthcare resources to manage outbreak peaks effectively.

While this persistent-homology-based time series analysis and clustering provides valuable insights into the spatial-temporal dynamics of the COVID-19 pandemic, its application is not without limitations. As we set the embedding dimension as 2 in this study, the temporal information of the time series is collapsed across two-dimensional phase space reconstruction plots. Indeed, the embedding dimension should be large enough that the state-space is sufficiently large to fully capture the range of patterns present in the data^51^, especially when longer time series are analyzed. From the extended analysis of the data from the pandemic’s onset to June 1, 2024, shown in Figs. S9 to S12 in Supplementary Information, not only is the computational time much increased, the quality of phase space reconstruction also degrades modestly. Hence, future works should consider partitioning the time series into smaller time window sizes for phase space reconstructions, increasing the embedding dimension, or employing complex network approaches^52^ for conducting nonlinear time series analysis of COVID-19 cases data.

Additionally, this study’s results reinforce the importance of data quality and preparation in epidemiological analysis. The effectiveness of different rolling averages in smoothing the data^53^ demonstrates the impact of data processing techniques on the outcome of topological analyses. Choosing appropriate smoothing parameters revealed more accurate and meaningful patterns in the COVID-19 case data, which could be obscured by noise and daily reporting anomalies. This emphasizes the need for careful data curation and preprocessing in epidemiological studies, particularly when employing advanced analytical methods like topological data analysis. The insights gained from this study contribute to a deeper understanding of the COVID-19 pandemic in Malaysia and offer valuable lessons for global public health surveillance and response systems in the face of future infectious disease outbreaks.

## Electronic supplementary material

Below is the link to the electronic supplementary material.

Supplementary Material 1

## Data Availability

The COVID-19 daily case data can be obtained from the open-source data of the Ministry of Health, Malaysia at https://github.com/MoH-Malaysia/covid19-public.
