# Supplementary material for: Nonlinear time series analysis of state-wise COVID-19 in Malaysia using wavelet and persistent homology
Source: Sci Rep. 2024 Nov 11;14:27562. doi: 10.1038/s41598-024-79002-0 (PMC11555112; doi:10.1038/s41598-024-79002-0)
Supplement: Supplementary file 1 — Supplementary Material 1 [file 41598_2024_79002_MOESM1_ESM.pdf]

Supplementary Information for "Nonlinear time series analysis of state-wise COVID-19 in Malaysia using wavelet and persistent homology"

Piau Phang*, Carey Yu-Fan Ling, Siaw-Hong Liew, Fatimah Abdul Razak, Benchawan Wiwatanapataphee

* pphang@unimas.my

Contents

Daily and cumulative cases throughout the study period; Geographical map of study area 2

Complete results on phase space reconstructed and persistence diagram for time series with 3
- 7-day rolling average ………………………………………………………………………. 3
- 15-day rolling average ……………………………………………………………………… 6

The hierarchical clustering on principal components 9

The extended analysis of COVID-19 data from the onset of the pandemic to June 1, 2024 10
- Time series plot ………………………………………………………………………. 10
- Phase space reconstruction plot ……………………………………………………… 11
- Persistence diagram ………………………………………………………………… 12
- Wasserstein distance and dendrogram …………………………………………….. 13

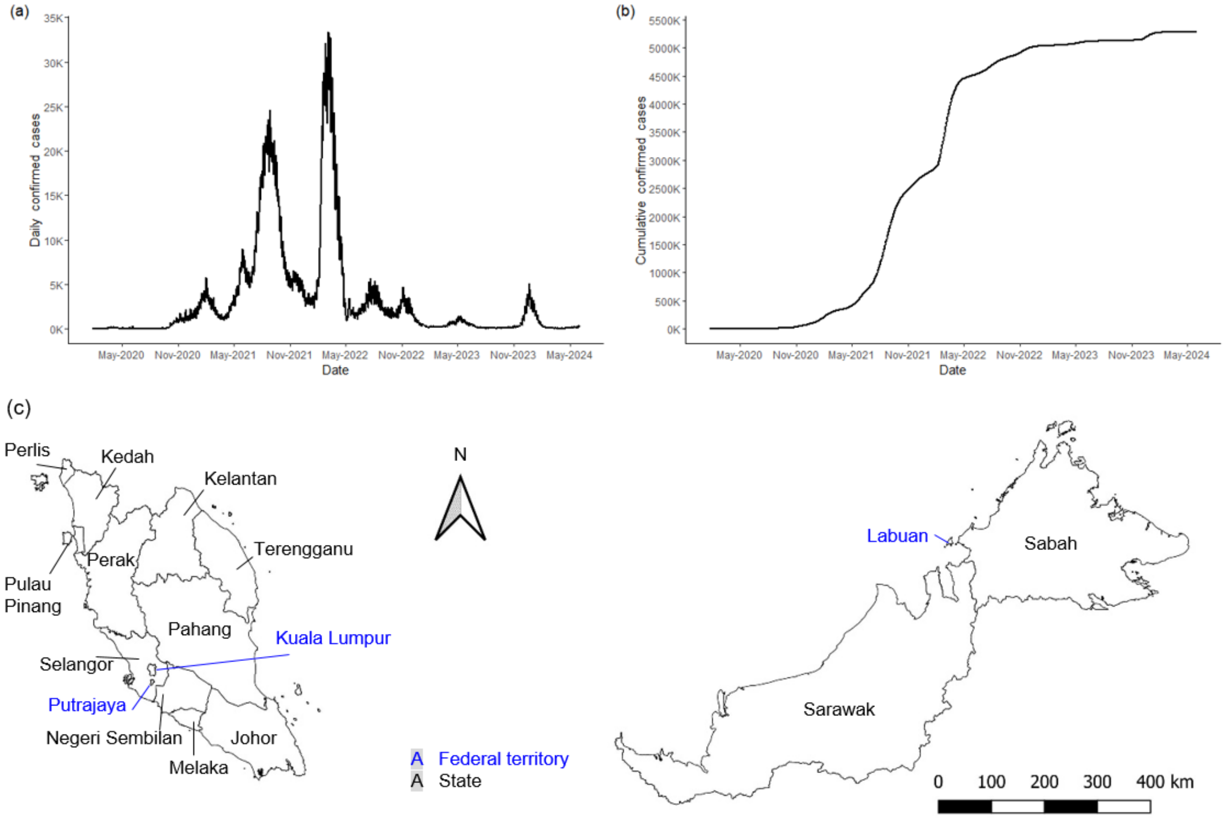

Figure S1. Line charts of (a) daily, (b) cumulative COVID-19 newly confirmed cases in Malaysia from 25 January 2020 to 1 June 2024. (c) Map of 13 states and 3 federal territories of Malaysia.

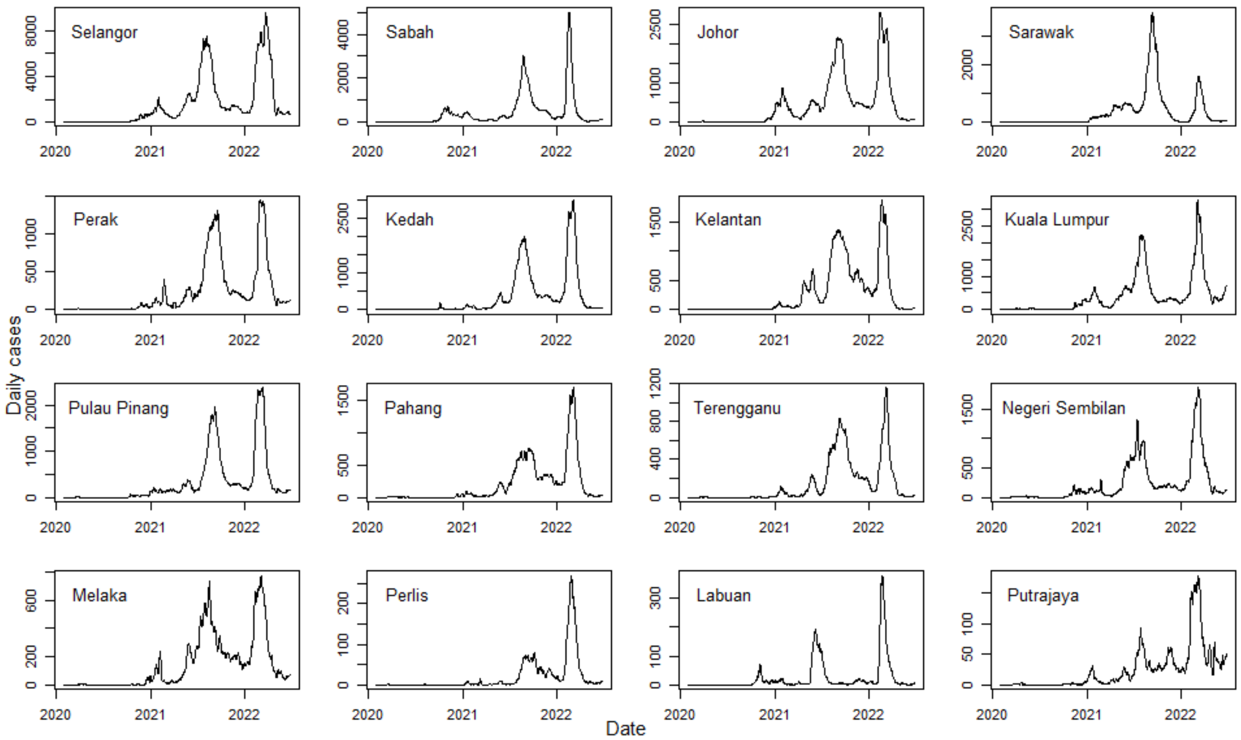

Figure S2. 7-day rolling average of state-wise COVID-19 daily confirmed cases from 25 January 2020 to 30 June 2022.

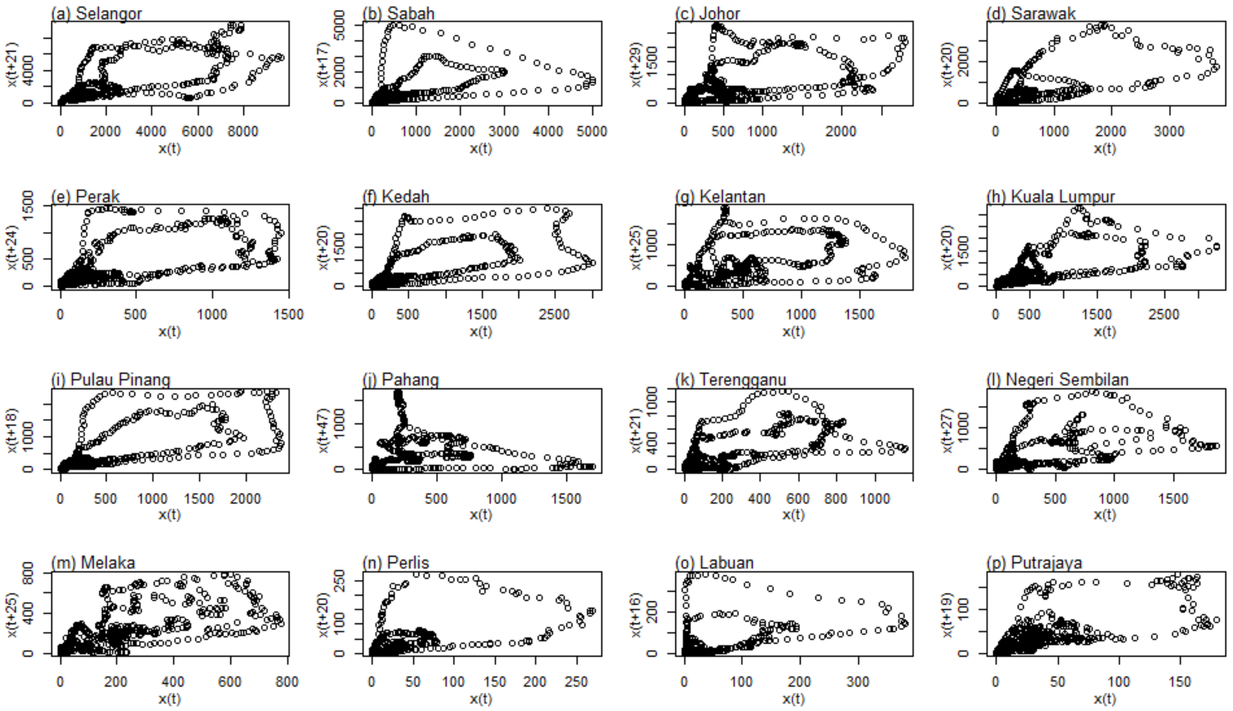

Figure S3. The phase space reconstruction plot for state-wise COVID-19 daily cases time series with a 7-day rolling average in Fig. S2. As the time series in Fig. S2 has a minimum of two peaks, we expect the phase space reconstructed to display one outer cycle and at least one inner cycle. However, such cycles are invisible, especially for (m) Melaka and (p) Putrajaya. Also, the phase space reconstructed for (c) Johor, (j) Pahang, and (l) Negeri Sembilan only partially preserve the structure of the time series. This reflects that the 7-day rolling average may still not be able to reduce the noise in the time series data for an optimal phase space reconstruction.

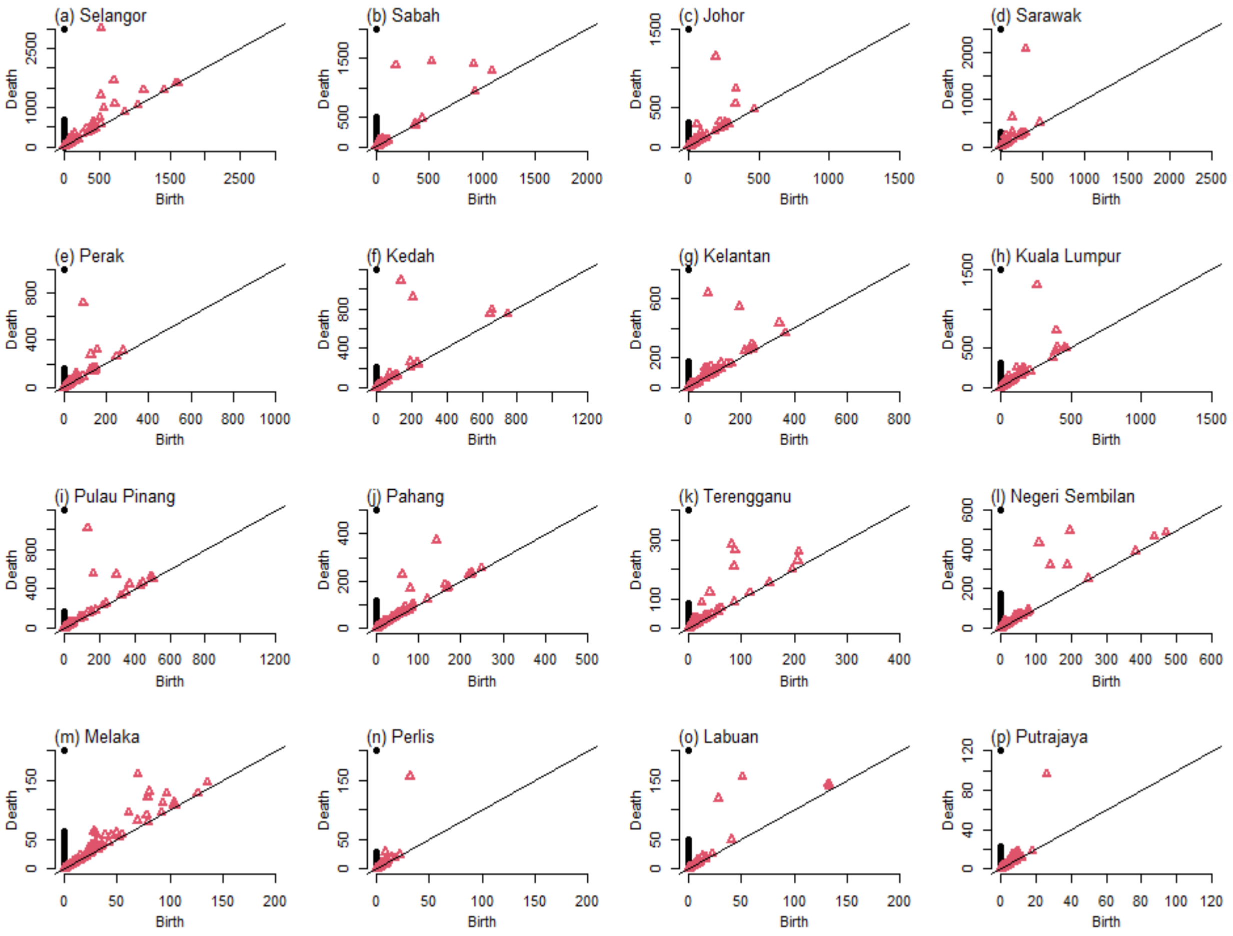

Figure S4. The persistence diagram for state-wise COVID-19 daily cases time series with a 7-day rolling average in Fig. S2. Due to practically bad phase space reconstructed in Fig. S3 for some states, only half of the persistence diagrams plotted above produced two significant topological loops (see the red triangles that are positioned further from the diagonal line). They include (b) Sabah, (d) Sarawak, (f) Kedah, (g) Kelantan, (h) Kuala Lumpur, (i) Pulau Pinang, (n) Perlis, and (o) Labuan.

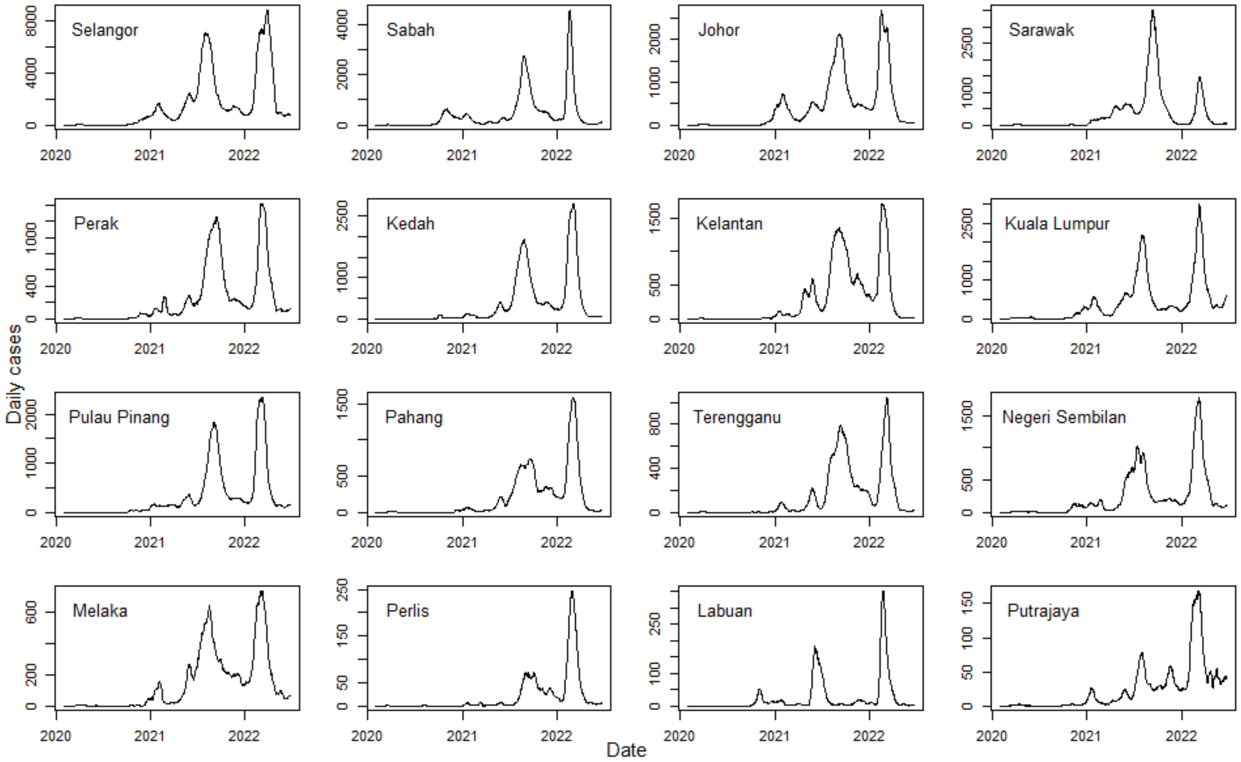

Figure S5. 15-day rolling average of state-wise COVID-19 daily cases from 25 January 2020 to 30 June 2022.

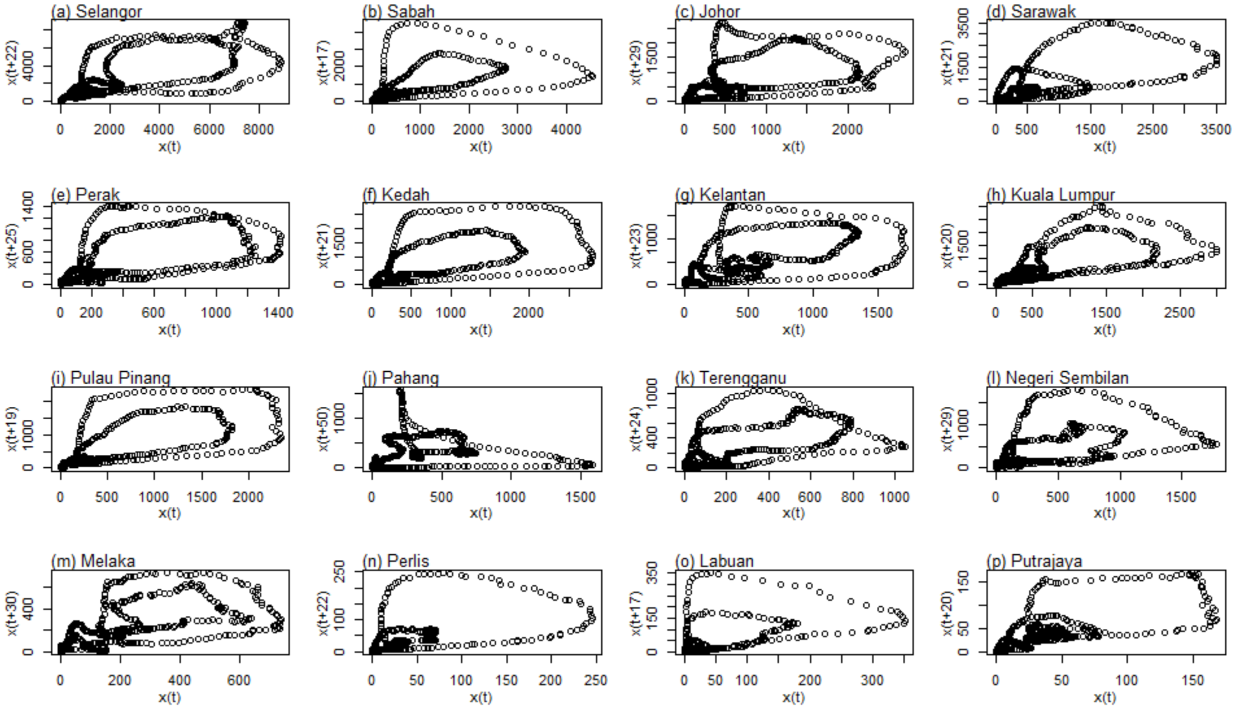

Figure S6. The phase space reconstruction plot for state-wise COVID-19 daily cases time series with 15-day rolling average in Fig. S5. Overall, the structure is practically well-preserved compared to their corresponding plot in Fig. S3. Also, one outer cycle and one inner cycle representing the respective Delta and Omicron waves are more visible, especially for (l) Negeri Sembilan and (m) Melaka.

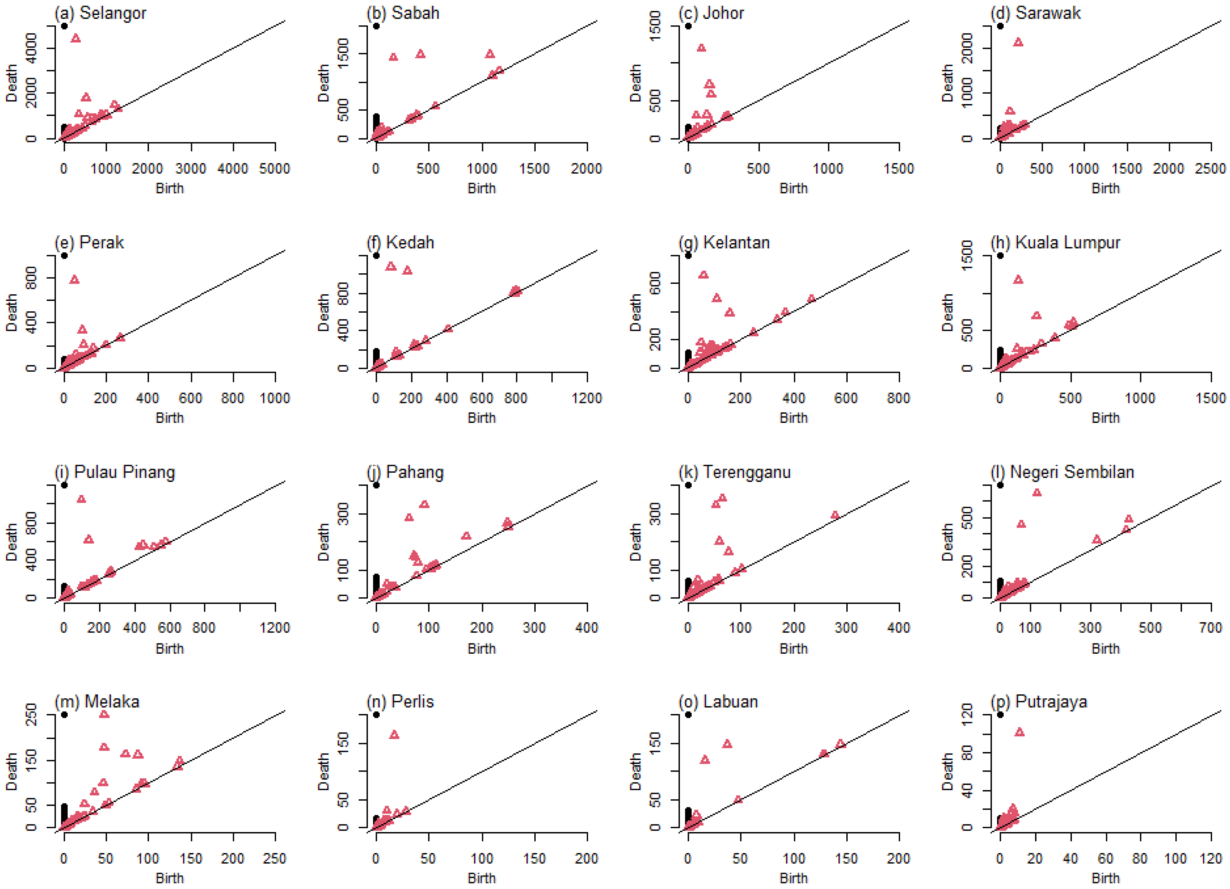

Figure S7. The persistence diagram for state-wise COVID-19 daily cases time series with a 15-day rolling average in Fig. S5. Almost all persistence diagrams in this figure display two significant topological loops (see the red triangles that are positioned further from the diagonal line), except for (n) Perlis and (p) Putrajaya as the peak of their second-largest waves are relatively low.

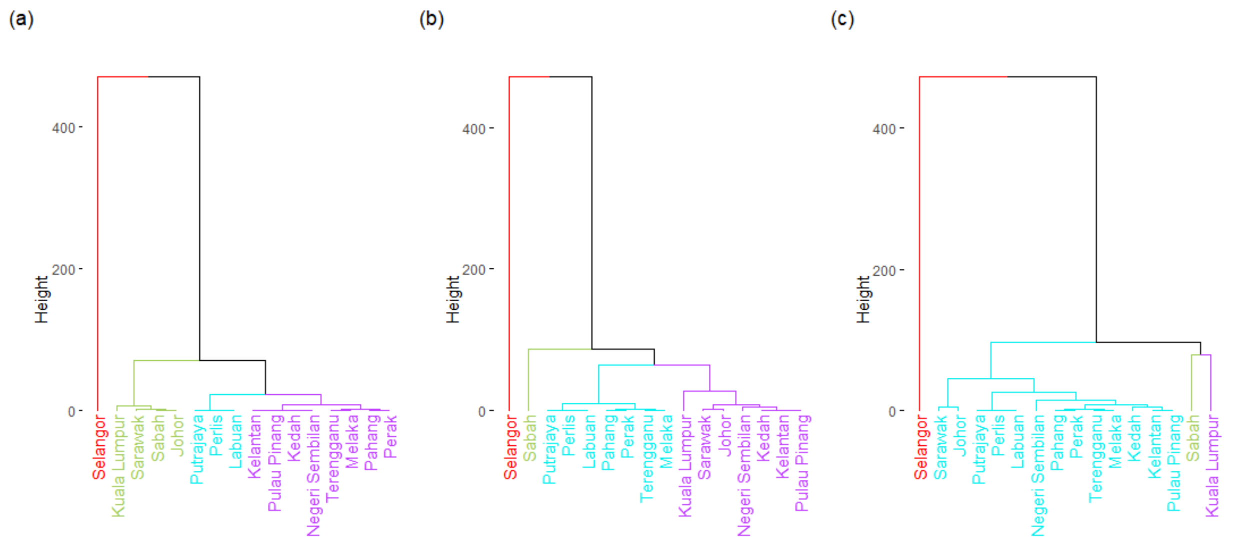

Figure S8. The hierarchical clustering on (a) principal component (PC) 1, (b) PC2, and (c) PC3 for state-wise COVID-19 daily cases time series with a 15-day rolling average in Fig. S5. All these hierarchical clusters are colored by setting the number of cluster to four to match the four clusters identified in Fig. 8(b) in the main text.

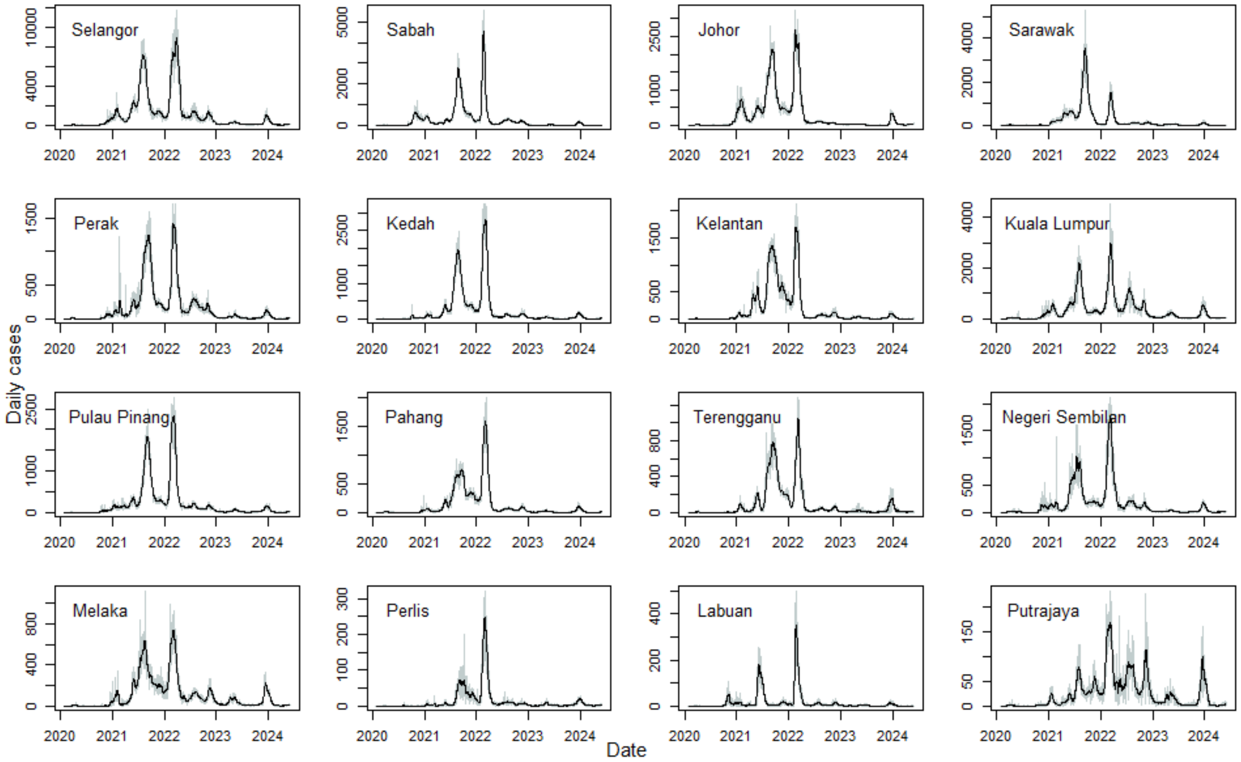

Figure S9. The raw (in grey) and 15-day rolling average (in black) of daily confirmed cases of COVID-19 in 13 states and 3 federal territories of Malaysia from the onset of the pandemic to June 1, 2024.

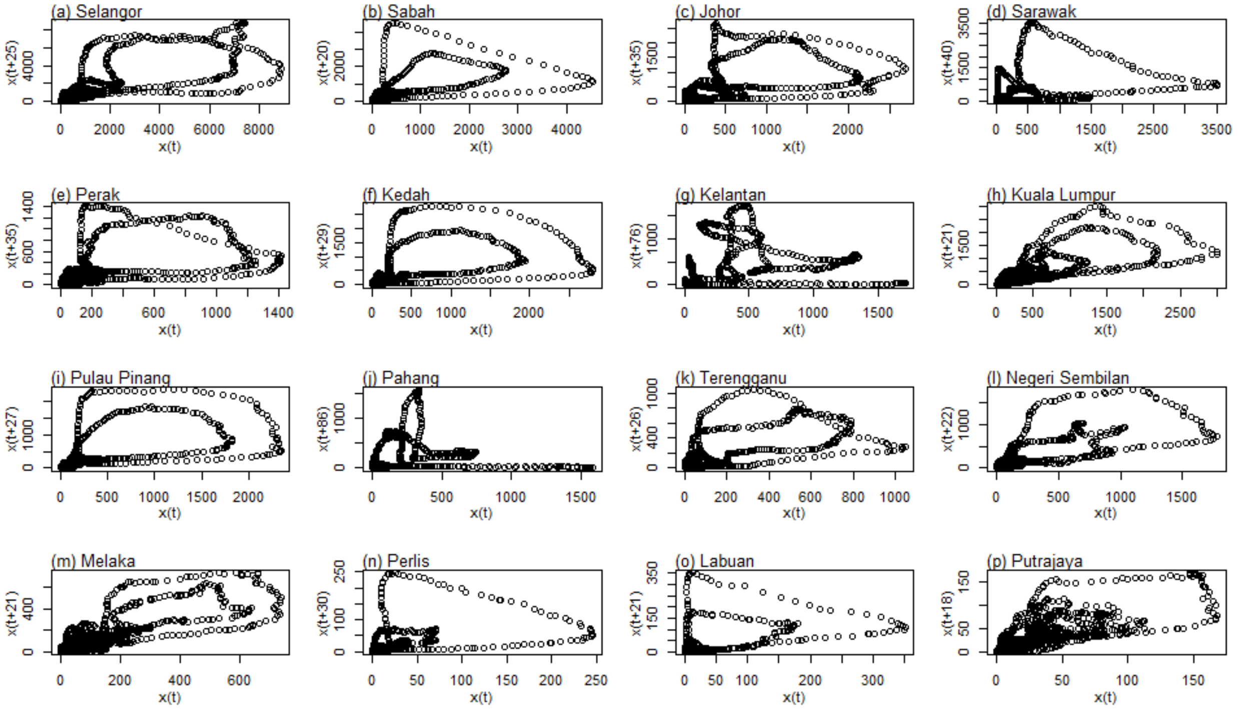

Figure S10. The phase space reconstruction plot for state-wise COVID-19 daily cases time series with a 15-day rolling average for the study period from the onset of the pandemic to June 1, 2024.

We expanded our COVID-19 data analysis to cover the period from the onset of the pandemic to June 1, 2024 (the latest available data at the time this manuscript is being revised). Figure S9 presents line charts depicting the case numbers for each state and its 15-day rolling average, while Fig. S10 shows their respective phase space reconstruction plot. As the time span for this extended analysis covers a 1590-day period, compared to 888 days in the previous analysis (see Fig. S6), the computational time becomes much longer than previous analysis. Also, the optimal time delay is generally larger than the corresponding time delay in Fig. S6, except for Negeri Sembilan, Melaka, and Putrajaya which have lower population sizes. A large time delay often indicates a more unstable and oscillatory behavior in a time series. For Kelantan and Pahang, their optimal time delay is exceptionally large. The appearance of one outer cycle and one inner cycle that represent the respective Delta and Omicron waves becomes not clearly visible in these two states' phase space reconstruction plots (see Figs. S10(g) and (j)). Also, when the time span is large, more points appear at the bottom left corner of the plot, especially for Selangor (Fig. S10(a)), Kuala Lumpur (Fig. S10(h)), Melaka (Fig. S10(m)), and Putrajaya (Fig. S10(p)). This implies that the time series in this extended analysis may require a proper partitioning technique not covered in this study to produce a better reconstruction plot.

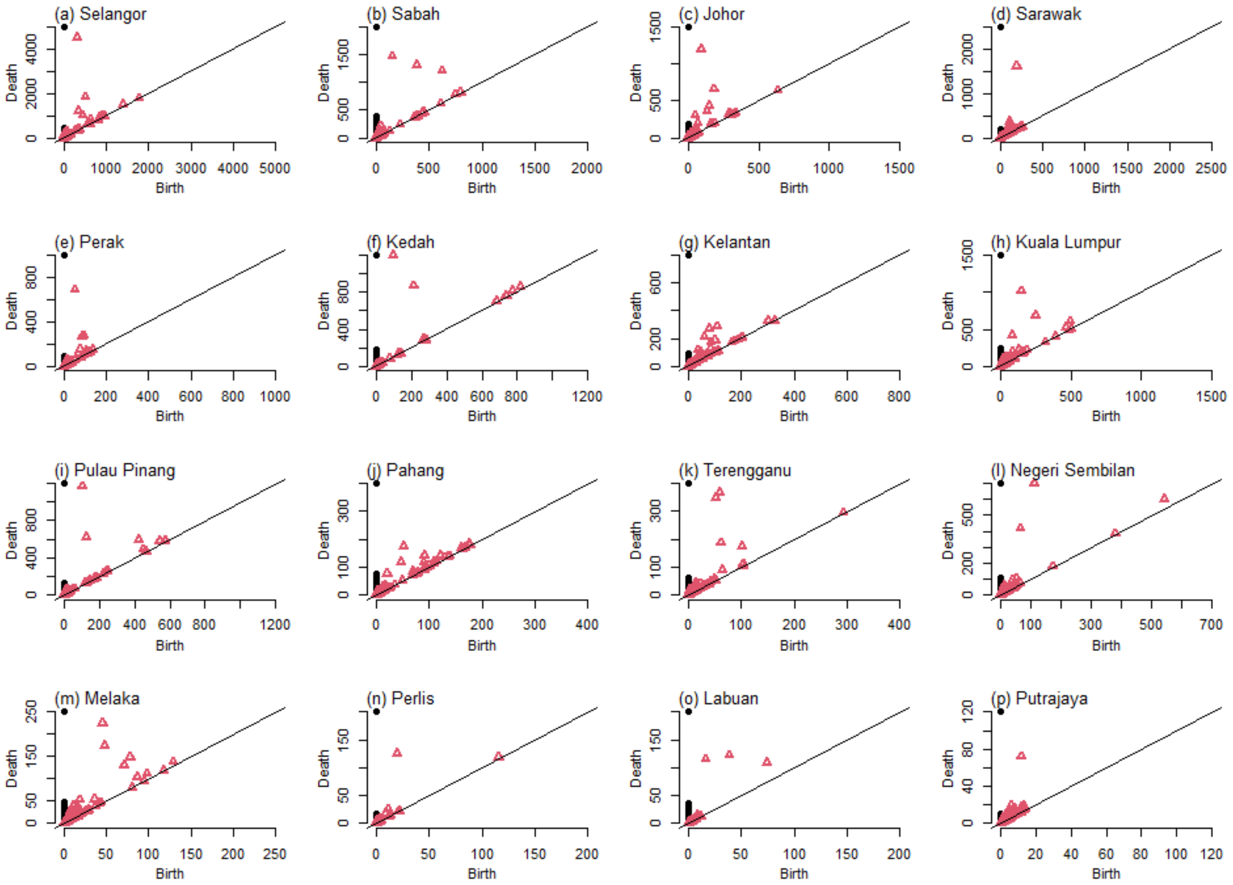

Figure S11. The persistence diagram for state-wise COVID-19 daily cases time series with 15-day rolling average in Fig. S9. All persistence diagrams in this figure produce significant topological loops (see the red triangles that positioned further from the diagonal line) which are almost identical to those in Fig. S7, except for (g) Kelantan and (j) Pahang as their phase space reconstruction plots are not practically well constructed in this extended analysis with longer time span.

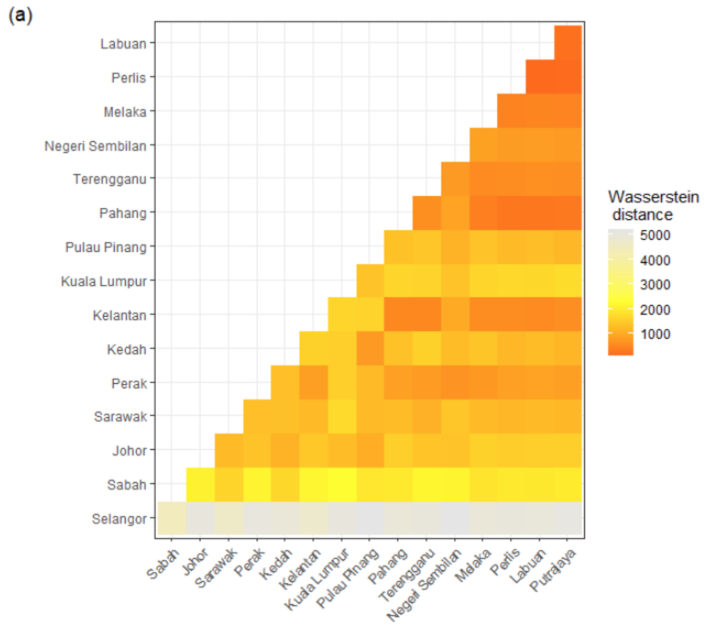

Figure S12. (a) Wasserstein distance between persistence diagrams for all 13 states and 3 federal territories in Fig. S11. (b) The dendrogram of 13 states and 3 federal territories resulting from hierarchical clustering of topological features extracted from their respective persistence diagram in Fig. S11. The results in this extended analysis generally agree well with those shown in Fig. 8 in the main text, except that five clusters may be more suitable for hierarchical clustering.
